# Thinking Aloud or Screaming Inside: Exploratory Study of Sentiment Around Work

**DOI:** 10.2196/30113

**Published:** 2022-09-30

**Authors:** Marzia Hoque Tania, Md Razon Hossain, Nuzhat Jahanara, Ilya Andreev, David A Clifton

**Affiliations:** 1 Institute of Biomedical Engineering Department of Engineering Science University of Oxford Oxford United Kingdom; 2 School of Information System Queensland University of Technology Brisbane Australia; 3 Department of Psychology University of Dhaka Dhaka Bangladesh; 4 School of Engineering and the Built Environment Anglia Ruskin University Cambridge United Kingdom; 5 Oxford Centre for Advanced Research (OSCAR) University of Oxford Suzhou China

**Keywords:** work-related mental health, sentiment analysis, natural language processing, occupational health, Bayesian inference, machine learning, artificial intelligence, mobile phone

## Abstract

**Background:**

Millions of workers experience work-related ill health every year. The loss of working days often accounts for poor well-being because of discomfort and stress caused by the workplace. The ongoing pandemic and postpandemic shift in socioeconomic and work culture can continue to contribute to adverse work-related sentiments. Critically investigating state-of-the-art technologies, this study identifies the research gaps in recognizing workers’ need for well-being support, and we aspire to understand how such evidence can be collected to transform the workforce and workplace.

**Objective:**

Building on recent advances in sentiment analysis, this study aims to closely examine the potential of social media as a tool to assess workers’ emotions toward the workplace.

**Methods:**

This study collected a large Twitter data set comprising both pandemic and prepandemic tweets facilitated through a human-in-the-loop approach in combination with unsupervised learning and meta-heuristic optimization algorithms. The raw data preprocessed through natural language processing techniques were assessed using a generative statistical model and a lexicon-assisted rule-based model, mapping lexical features to emotion intensities. This study also assigned human annotations and performed work-related sentiment analysis.

**Results:**

A mixed methods approach, including topic modeling using latent Dirichlet allocation, identified the top topics from the corpus to understand how Twitter users engage with discussions on work-related sentiments. The sorted aspects were portrayed through overlapped clusters and low intertopic distances. However, further analysis comprising the Valence Aware Dictionary for Sentiment Reasoner suggested a smaller number of negative polarities among diverse subjects. By contrast, the human-annotated data set created for this study contained more negative sentiments. In this study, sentimental juxtaposition revealed through the labeled data set was supported by the n-gram analysis as well.

**Conclusions:**

The developed data set demonstrates that work-related sentiments are projected onto social media, which offers an opportunity to better support workers. The infrastructure of the workplace, the nature of the work, the culture within the industry and the particular organization, employers, colleagues, person-specific habits, and upbringing all play a part in the health and well-being of any working adult who contributes to the productivity of the organization. Therefore, understanding the origin and influence of the complex underlying factors both qualitatively and quantitatively can inform the next generation of workplaces to drive positive change by relying on empirically grounded evidence. Therefore, this study outlines a comprehensive approach to capture deeper insights into work-related health.

## Introduction

### Background and Motivation

The economic growth of the human civilization, along with our understanding of medical science in combination with the recent technological advancements, has brought us to question how to design the workforce management system in the era of Industry 4.0. The image of occupational health has been gradually shifting from its strong association with workplace injuries to work-related ill health. Among the European nations, most of the UK workforce trust that their health or safety is not at risk because of their workplace [1]. However, much remains to be done to achieve more efficiency in workplace management as millions of workers are still experiencing work-related illnesses every day [1,2].

According to the recent Health and Safety Executive (HSE) report covering the period from 2020 to 2021, 1.7 million workers experienced a work-related illness, approximately half of which were due to stress, depression, or anxiety [1]. In the prepandemic report, >800,000 workers experienced mental health issues, whether a new presentation or a long-standing condition, because of the workplace, resulting in the loss of 17.9 million working days. According to the Labour Force Survey (LFS) studies, workload, extreme deadlines, excessive responsibility, absence of adequate managerial support, violence, threats or bullying, and changes at work such as reformation are estimated to be the main reasons behind such poor mental health [2]. Although factors vary from industry to industry, in terms of occupation, professional occupations (examples include, but are not limited to, scientists, engineers, programmers, health workers, and teaching and educational professionals) show higher levels of stress compared with all jobs.

If work-related mental health was not already a concern, COVID-19 has added more dimensions to it. The increase in depression among adults in the United Kingdom during the pandemic has been well reflected in the latest Office for National Statistics report [3]. Although the HSE presented self-reported work-related stress, depression, and anxiety from the LFS study, the finding echoes mental health figures among the adult population in the United Kingdom [4]. The HSE report also quantifies how work-related well-being is affected among different age groups and genders. The gender difference in mental disorders such as depression and anxiety is fairly common, as reported by the World Health Organization [5]. Therefore, formulating a resilient workplace would require a comprehensive understanding of the age and gender disparity in the loss of working days to circumvent human as well as algorithmic bias.

By their very nature, mental health issues and well-being are difficult to measure, and the HSE has 2 different data sources from which analyses are conducted—none of which record real-time data. Moreover, work-related new or long-standing ill health as a consequence of long or irregular working hours, stress, anxiety, panic, hidden or unrealistic expectations, job insecurity, instability in the job market, societal pressure, rat race, and macho culture, which contributes to an unhealthy lifestyle, often gains visibility in the form of eating disorders, irregular or insufficient sleep, and addiction.

To probe beneath symptoms on the surface by separately tagging each attribute with the associated influence as well as contributing factors adds more complexity when exposing such vulnerability may result in job loss or at least an obstruction to career progression. Hence, it is understandable that approximately 15% of working adults show indications of symptoms of an existing mental health condition [6] as >300,000 people each year lose employment because of mental health problems [7]. By contrast, technological advancement has opened the door to harnessing the power of wearable sensors, the Internet of Things, and artificial intelligence (AI) to gather richer insights on general mental health issues among the population as well as industry-specific personalized circumstances (within and because of the workplace).

### State-of-the-art Technological Support

Exploring recent quartile 1 and 2 journals and top conference publications using search words such as “mental health AND artificial intelligence,” “mental health AND decision support system,” “mental health AND mHealth,” and “mental health AND mobile apps” through Google Scholar, PubMed, PsycArticles, ScienceDirect, and Psychology and Behavioral Sciences Collection search engines, we discovered that the broader domain in the literature covers the area of severe mental illness such as schizophrenia [8-11]; anxiety disorders [12] such as posttraumatic stress disorder [13]; developmental disorders such as attention-deficit/hyperactivity disorder [14]; and, of course, disorders that are often not recognized as an outcome of occupational health hazards affecting the everyday quality of life [15,16]—however, neglecting these early indications may even trigger suicidal tendencies [17-19].

Similar to physical health, state-of-the-art investigations on mental health aspects are mainly geared toward screening, diagnosing, and phenotyping purposes [20-23]. However, web-based [24,25] and mobile-based interventions [26-29] are not far behind.

A wide range of technology-enabled support has been investigated in the literature, starting from simple tools such as traditional SMS text messages [30] to cutting-edge technology such as AI-enabled chatbots [31]. Mental health and well-being apps have millions of installations worldwide [32], which makes the demand for such apps evident; however, very few studies have been conducted to verify and validate the capabilities of these apps to bring positive changes in users’ mental well-being.

With or without smartphone integration, recent advancements in AI have brought some commercial successes, such as Babylon [33], Quarte [34], Lyra [35], Ginger [36], Woebot [37], and BioBeats [38], to support health and well-being, backed up by collaborative research with world-leading universities. Most of these available apps on the market consist of AI-enabled chatbots and use natural language processing (NLP), such as the AI-driven personalized triage and symptom checker tool by K [39] and CBT by Woebot.

Within the mental health area using data-driven approaches, a New York–based start-up, Spring Health [40], has developed an AI-driven personalized triage and symptom checker for the mental health of employees, whereas a London-based company called BioBeats provides an AI-enabled intervention for stress management. BioBeats developed an intelligent app with a business-to-business model that provides a well-being score based on physiological data (sleep duration and quality and heart rate variability), psychological data (mood journaling), and neuropsychological data (brain function tests) collected through their BioBase mobile app and BioBeam wearable. Tools such as BioBeats can enable their users to gain insights into personal mental health. Such scientifically validated well-being tools can be thought of as company-provided perks. By gathering aggregated and real-time but anonymized data from employees using the BioBeats platform, the employer can track the well-being of their staff and take actions such as providing tailored support when needed. Continuous monitoring can help understand and even quantify whether a change in the company’s policy is compromising the well-being of the employees.

### Sentiments on Social Media Platforms: Related Works

Microblogging today has become a popular communication tool among internet users. Millions of messages appear daily on popular websites that provide services for microblogging, such as Twitter and Facebook. This is due to the nature of microblogs, on which people post real-time messages about their opinions on a variety of topics, discuss current issues, complain, and express positive sentiments about their daily lives. Owing to the free format of the messages and easy accessibility of microblogging platforms, internet users tend to shift from traditional communication tools (such as traditional blogs or mailing lists) to microblogging services. Therefore, the use of social media has become an integral part of daily routine in modern society.

In terms of the choice of microblogging platform, privacy concerns have been observed in the literature [41]. The type of self-disclosure also has an impact on the use of such social media [41]. It has been seen that the users of Twitter have high self-disclosure. People who like to bond socially prefer using Facebook, as suggested by Shane-Simpson et al [41].

Some of the positive use cases of social media include pandemic studies. Twitter has been used to explore diverse issues such as sentiment alteration [42], lockdown [43], sentiment around hospital care management [44], vaccination [45], and remote working [46]. The use of social media for health and well-being research is common, but it is mostly limited to participant recruitment. However, in a study in San Diego, the health outcomes of the local people were observed using tweets that measured self-rated mental health, sleep quality, and heart disease [47].

Previously, data from social media profiles of US military personnel have been used by Bryan et al [48] to find predictors of suicide. Analysis of the tweets revealed various aspects of their lives along with the triggering factors in stressful situations, such as health issues, maladaptive or avoidant coping strategies, emotional state, and cognition. It has been observed by Bryan et al [48] that the manner of posting can differ between suicidal and nonsuicidal users. A pattern of posts was also observed as the “trigger” posts increased before suicide following negative emotional posts. Suicidal people who posted about maladaptive coping frequently were followed by a few negative emotional posts [48].

Among the working adults in the United Kingdom, suicide is more prevalent in the construction industry [49]; the use of drugs, alcohol, marijuana, and other substances is also common among this workforce [50]. Although not specified for construction workers, using data from Twitter-based advertisements, the reasons for using marijuana and the characteristics of marijuana users were explored in the reported article [51].

Several studies have investigated the psychological rationale for the temptation to share on social media and the mental profile of those who share on social media and to what extent [52]. However, a more critical exploration with clinical validation is required. By contrast, people share on social media to maintain social connections even if it is not a pathological need.

Using adolescent population data from thousands of people aged 14 years, Kelly et al [53] attempted to investigate whether mental health is linked to the use of social media. The study concluded that a positive correlation exists between the use of social media and depressive symptoms. Other factors that influence the use of social media are poor sleep, low self-esteem, poor body image, and harassment on the internet. These factors are the underlying symptoms of depression as well. Social media use can result in increased perceived social isolation [54]. Too much social media use can also cause social media fatigue, affecting psychological well-being [55].

Although the aforementioned studies show the psychological aspect of why people share on social media, the underlying rationale of social media use by individuals, as well as the impact, can be more complex [56]. Moreover, technological tools such as “Gamification” using neurological hacks can influence social media use, as observed by Bell et al [57].

Social media could be an innovative tool for interventions. However, the impact can go either way as Weinstein [58] observed a seesaw effect of social media while exploring variables such as relational interactions, self-expression, interest-driven exploration, and browsing.

Interestingly, the deepest insights within the context are owned by the social media industry, and third parties in the name of “personalization” and “enhanced experience,” which have the ability to trigger social media addiction, are yet to be extensively studied.

The “Big Five personality traits” (ie, neuroticism, agreeableness, conscientiousness, openness, and extroversion [59]) can be traced from the data obtained from social media using digital footprints [60]. Estimation and quantification of personality traits can support the use of social media for the greater good beyond the personalization feature of social media itself. It is important to associate the understanding of the psychological need to “share” emotions on internet-enabled platforms with the machine-learned quantified measurement of sentiments before taking actions based on an under- or overestimation of our emotions.

### Research Aim

This paper presents an investigation into the methods and tools to understand and analyze how sentiments around work are expressed, which can be further used to flag such issues and design effective interventions. This paper presents a literature review in the Introduction section exploring both reported articles and available state-of-the-art technological support. This paper also provides criticism on vulnerable aspects. The key contributions of this study include the following: (1) to the best of our knowledge, this is the first work performing sentiment analysis and topic detection concentrating on work-related sentiments comprising both pandemic and prepandemic tweets; (2) we collected a large data set based on a hybrid approach for keyword search; (3) we created a labeled data set; and (4) the preprocessed complete data set, as well as the labeled data set on work sentiment, will be made available, which may create opportunities for further studies.

The rest of the paper is organized as follows. The Methods section presents the methods used to critically investigate work-related sentiments on Twitter, followed by the analysis of the results in the Results section. This paper includes an in-depth discussion in the Discussion section highlighting the intertwined vicious cycle of physical and mental health and also the ethical concerns regarding the use of technology. This paper also discusses a conceptual framework for AI-enabled mental health support systems for the workforce. Finally, we discuss the study’s limitations and scope for improvement while concluding the paper.

## Methods

### Microblogging Platform

This study explores work-related sentiments on Twitter. Twitter, with >319 million monthly active users, has now become a goldmine for researchers, organizations, and individuals to survey public health trends because of the nature of the data source. Twitter allows developers to fetch data (ie, tweets) from its archive using an application programming interface. On the basis of the literature, it is anticipated that AI-enabled tools such as sentiment analysis will help us better understand how people talk about and feel with respect to specific health topics or conditions—in this case, mental health–related issues linked to work—in real time.

### Data Fetching and Processing

In this study, we used the Python-based library SNScrape (Scraper for Social Networking Services) [61] to fetch tweets using the defined primary keywords from Textbox 1. We used these keywords to formulate the keywords for fetching tweets according to the procedure described by Edo-Osagie et al [62]. The search query, dated from January 1, 2018, to December 31, 2021, resulted in >1 million tweets containing at least one keyword either as a hashtag or in the tweet body.

**Primary keywords used to form the keywords for fetching tweets.**

**Primary keywords**

- Nouns: “work,” “job,” “industry,” “labor,” “labor,” “office,” “workplace,” “occupation,” “employment,” “employee,” “employer,” “corporate,” “company,” “enterprise,” “profession,” “meet deadlines,” “cubicle,” “workplace”
- Adjectives: “stress,” “anxiety,” “depression,” “inefficiency,” “multitasking,” “breaking point,” “overload,” “burnout,” “stressed out,” “under pressure,” “demoralization,” “depressed mood,” “horrific,” “shocking,” “dangerous,” “problematic,” “undesirable,” “sad,” “struggle,” “stressed,” “depressed,” “ill,” “illness,” “mental strain,” “best lucky,” “bepositive,” “bethet united,” “get in,” “vibes,” “ftw,” “accomplished,” “let’s get it done,” “mic dropped alhamdulillah,” “agreed,” “happy,” “good,” “enjoyment,” “proud,” “satisfaction,” “bad,” “unlucky,” “sorry,” “bad,” “worse,” “bad,” “hurt,” “tiring,” “long-day,” “boring,” “exhausted,” “hopeful,” “2ndhome,” “standwith,” “godspeed,” “more to follow”
- Phrases: “overwork,” “wage slave,” “ironwork,” “mental health,” “congratulations office,” “work life,” “workoholic,” “worklifebalance,” “job strain,” “bad day at the office,” “when going gets tough workaholic gets going,” “comeback,” “back to drawing board,” “work-life balance”

### Keyword Selection Process

#### Hybrid Steps

The Twitter search uses words to find tweets relevant to the quest. These words or parts of sentences are called keywords. Proper keywords help to find and fetch tweets that are efficient and accurate to the context [63]. Therefore, the selection of keywords is a crucial step in fetching tweets. We followed a 2-fold process to select the keywords.

#### Phase 1: Keyword Collection From a Panel

First, a panel of working professionals was formed to estimate the linguistic diversity in the expression of emotions regarding the workplace and work. There were 7 members in the panel, and they were proficient users of social media, had a successful background in tertiary education, and were in a responsible position at work. The responses were collected using Google Forms. We received responses from a diverse range of industries and professions, including software engineers, corporate officers, and academics. The panel also involved experts from work rights organizations. The tenure of work experience was also recorded as background information; however, it was not used in the data analysis because of the size of the panel.

In this phase, the panel members were asked how they articulated work-related sentiments on social media, specifically on Twitter. The responses were recorded anonymously. The panel members were asked about their linguistic preference to denote work and the workplace (inclusive of words and phrases with similar semantic meanings and similar sentiment-expressing adjectives, whether positive, negative, or neutral). We divided these words into 3 categories, which are displayed in Textbox 2.

Category 1, also denoted as nouns in Textbox 1, represents the semantic expression of “work,” manually identified by the natural language users (ie, the panel of working adults on Twitter). These keywords mean only something related to work. In contrast, category 2 aims to capture the core sentiment. The set of words in category 2 can be related to anything, including work and the workplace. However, adding an adjective to the synonyms of *work* and *workplace* means that the sentiment expressed is related to work.

For example, the words “office” or “employer” alone do not always express the sentiment regarding work life and can be used in many other ways, such as the address of a workplace or a job circular.

Moreover, the adjectives “struggle” or “good” alone can also be related to anything. However, to increase the search efficiency and fetch more contextual tweets, we searched for tweets that contained both “office” and “good” or “office” and “struggle.”

Furthermore, the participating professionals were from a particular region and were limited in number. Although there was diversity in their occupations, the words and phrases they used certainly cannot completely represent or match the words and phrases of millions of other professionals and employees. Therefore, we can obtain more possible forms of keywords by combining each word in category 1 with each word in category 2.

In a nutshell, the reasons behind creating category 1 and category 2 are that (1) to search tweets that contained work-based sentiment, we would need related words or synonyms to “work” or “workplace” (category 1) and words that expressed “sentiment” or “emotion” regarding these (category 2) and (2) to fetch more diverse and the highest number of tweets, we needed to pragmatically maximize all possible forms of keywords and, therefore, separating them into different categories, represented as rows in Textbox 1, helped us combine category 1 and 2 with each other and increase the number of keywords.

Subsequently, we also obtained some phrases from the professionals (category 3) that directly describe work-related sentiments, such as “wage slave” and “congratulations office.”

**Categories of words and phrases in Textbox 1 used to denote work and the workplace.**

**Categories of words and phrases in Textbox 1**

- Category 1: words and phrases implying work, the office, or the workplace
- Category 2: words and phrases signifying sentiments (can be positive, negative, or neutral)
- Category 3: phrases that explicitly mean sentiments regarding work, the office, or the workplace

#### Phase 2: Keyword Formulation Using Algorithms

Despite the careful choice of a panel of working professionals, there was a need for an enhanced set of keywords owing to human bias, spelling practices on social media, the internet, and work-related jargon. Moreover, we considered only English keywords, whereas there are thousands of languages in the world. Hence, the collected words and phrases can be limited, and there can be far more words and phrases used by millions of workers worldwide. Therefore, to generate more keywords, we used the Global Vectors for Word Representation (GloVe), which process billions of tweets and generate synonyms based on the context. The workflow in this phase is shown in Textbox 3.

GloVe is a well-known algorithm that uses billions of tweets to generate synonyms with a context. Therefore, in the second phase, we used GloVe to generate 5 synonyms for each word in categories 1 and 2 from Textbox 1. We considered all the words in category 1 and their generated synonyms as a prefix list and all the words in category 2 and their generated synonyms as a postfix list. These prefix and postfix lists contain unique words. For each word in the prefix list, we added all the words from the postfix list and generated the seed keyword list. Let us assume that the prefix list is (workplace, corporate) and the postfix list is (stress, good). Therefore, the seed keyword list is (workplace stress, workplace good, corporate stress, corporate good).

We used each item from the “seed keyword list” to fetch the seed tweets to define the final keywords. For each item on the list, we split the words based on space and searched for tweets that contained these words. For example, the item “workplace stress” in the aforementioned “seed keyword list” contains 2 words: “workplace” and “stress.” Therefore, we searched tweets that contained both “workplace” and “stress” in any sequence and any number.

Subsequently, we used these seed tweets and seed keywords to generate the final keywords using a powerful metaheuristic optimization algorithm (ie, the particle swarm optimization algorithm).

**Flow of phase 2.**

**Flow**

- For each word or phrase in category 1 and category 2, generate 5 synonyms
- Consider all words and phrases in category 1 and their synonyms as a prefix list
- Consider all words and phrases in category 2 and their synonyms as a postfix list
- To generate all possible seed keywords (eg, the keyword list is *KT*):
  - For each word or phrase in the prefix list: *Wpre*
    - For each word or phrase in the postfix list: *Wpst*
      - *KT.add(Wpre+’ ‘+Wpst)*
- Fetch seed tweets by using this *KT*
  - For each keyword in *KT*:
    - If the keyword contains more than one word:
      - Split the words
      - Fetch tweets that contain these words
- Use these seed keywords and fetched seed tweets in the particle swarm optimization algorithm to generate actual keywords

### Data Preprocessing

**Figure 1:**
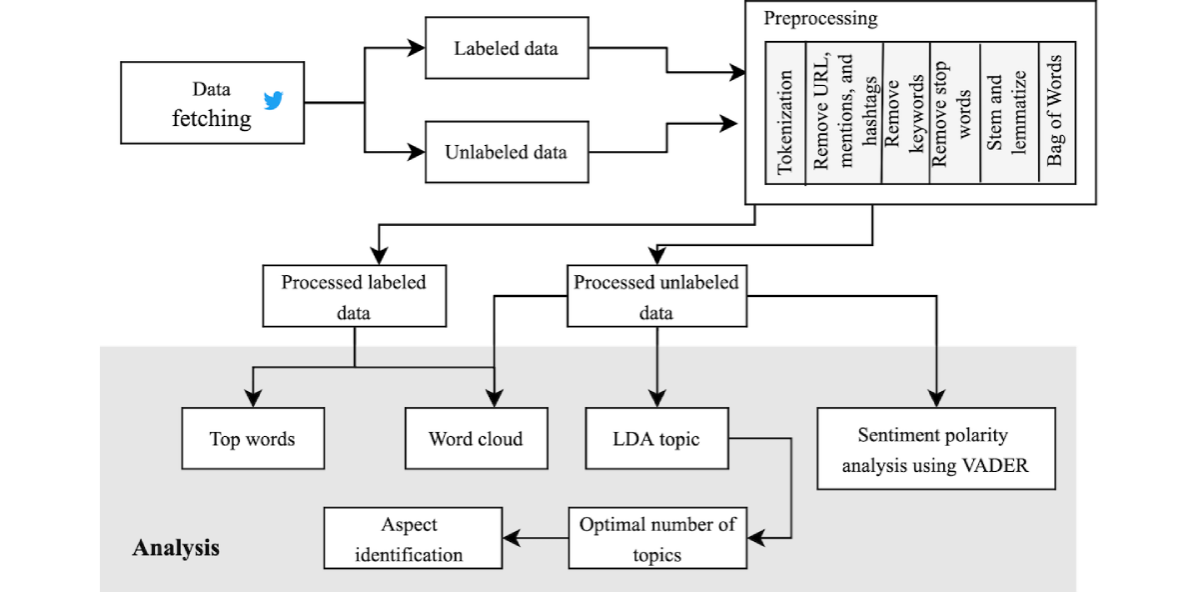
Preprocessing and analysis steps for both labeled and unlabeled data. LDA: latent Dirichlet allocation; VADER: Valence Aware Dictionary for Sentiment Reasoner.

The aim of preprocessing is to clear all the redundant and unnecessary content from the data and make them precise to find more accurate words that can help analyze the tweets. With this goal, the fetched tweets were systematically preprocessed (Figure 1) using a number of Python libraries such as Pandas, NumPy, and Natural Language Toolkit (NLTK) before initiating the analysis.

Each tweet was converted into lower case to easily identify the repetitive words and tokenize to convert the tweet into a list of words. The elimination of the selected keywords facilitated a better estimation of the frequency of words and easy spotting of the actual words used in expressing a sentiment. The contractions were expanded to their original forms. The data cleaning steps also included the removal of alphanumeric words, URLs, markup texts, mention words, stop words, hashtags, repeated characters, punctuation symbols, white spaces, and single characters from the tweets. Subsequently, normalization was achieved through lemmatization and stemming.

The purpose of this study was to inspect personal tweets, not organizational or commercial tweets. In an attempt to separate such tweets, more words were required to be eliminated, which are marked as advertisement words in Textbox 4. In addition, for simplicity of analysis, incomplete and misspelled words and unrecognizable shortened words had to be removed as well. The quality of the preprocessing step was verified through a qualitative inspection of random processed tweets. This inspection was carried out after each preprocessing step. The qualitative inspection also supported the recognition of meaningless words and advertising tweets.

**Advertising and meaningless words in the labeled data set.**

**Meaningless and advertising words**

- Meaningless words: *thi*, *wa*, *WA*, *rt*, and *ann*
- Advertising words: *click* and *coupon*

### Data Processing and Labeling

This paper presents an investigation into the tweets (Textbox 1) through the latent Dirichlet allocation (LDA) [64] model, using the Gensim (RARE Technologies Ltd) [65] library to explore issues discussed on Twitter and the Valence Aware Dictionary for Sentiment Reasoner (VADER) [66] model to estimate the sentiment of each tweet.

The investigation began with the identification of top words within the processed data set. The corpora of Gensim returned an ID term dictionary object, which subsequently created a corpus by converting each tokenized tweet into a word matrix. Later, the LDA model–generating function from the Gensim library used this dictionary and corpus and returned an LDA Model. This LDA Model consisted of a number of specific topics. Each topic contained a number of words along with their weight in the tweet. Using LDA Model, all the tweets were divided into several topics, which enabled us to understand the aspect of each tweet that can be representative of a broader classification.

**Figure 2:**
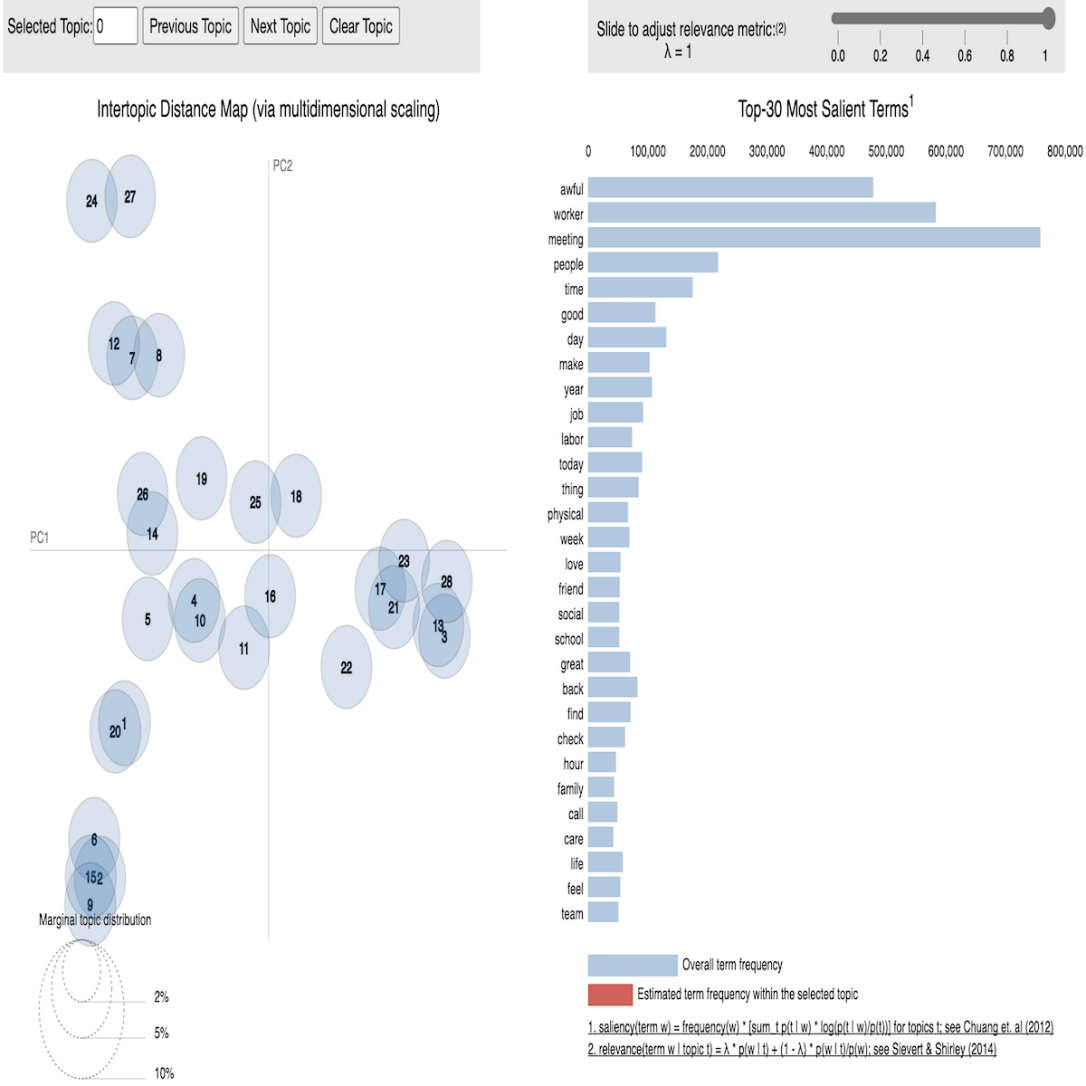
Distribution of the optimal number of topics with the top 30 salient words. PC1: principal component 1; PC2: principal component 2.

However, overlap among these topics was expected, which might hinder the identification of an aspect of a topic accurately. Hence, it was necessary to explore the optimal number of topics. Each LDA Model with a specific number of topics had a specific coherence. Coherence measures the score of semantic similarity between the contexts of multiple documents and, the higher the coherence value, the better the context-based topic distribution [67]. Therefore, the LDA Model with higher coherence has an optimal number of topics and, consequently, fewer overlapped topics. Moreover, the LDA Mallet wrapper on top of LDA provides better topic distribution and coherence and, therefore, we used the LDA Mallet wrapper to identify the optimal number of topics [68]. Afterward, we converted this optimized number of LDA Mallet topics into LDA Model topics for visualization, as shown in Figure 2. Such optimization was achieved through the iterative process of varying topics from 2 to 31. The coherence value for each iteration is shown in Multimedia Appendix 1.

Consequent to topic optimization, broader polarity of sentiments (ie, positive, negative, and neutral) was assigned with the help of the opinion dictionary library from NLTK. The aspect exploration process used in this study can also be observed in algorithm 1 (Textbox 5).

Next, a lexicon and rule-based model, VADER, was used to categorize the unlabeled tweets into our predefined 3 categories (positive, negative, and neutral) of sentiment polarity by analyzing each tweet. VADER considered the sentiment lexicon used in social media microblogs and generalized grammatical and syntactical aspect rules for identifying sentiment intensity. Moreover, it incorporated a human-centric approach combining qualitative analysis with empirical validation and experimental investigations, leveraging the wisdom of the crowd to calculate values based on the sentiment of a sentence. Furthermore, the performance of their algorithm with a lean lexicon and rule-based model was compared with other well-known and familiar sentiment analysis benchmarks [66], estimating a compound value ranging from −0.05 to +0.05 by considering the positivity and negativity of the tweet. If the sentiment value of the tweet was between −0.05 and +0.05, the tweet was neutral. A tweet with a value <−0.05 was negative, and a tweet with a value >+0.05 was considered positive. However, the lower the value, the higher the negative intensity, and the higher the value, the higher the positive intensity.

Reckoning the compound values and visualization, the percentage of each category aided us in determining which category had a greater number of tweets, whereas visualization of the compound values of each tweet helped us realize the fluctuations in values for each polarity.

In addition to the machine-assigned polarity of sentiments, this study included manual labeling of a subset of 3200 unprocessed random tweets as a pragmatic approach to further analyze positive, negative, and neutral tweets, with a higher chance to recognize juxtaposition of sentiments and sarcasm. As the labeling was conducted before preprocessing, exclamatory marks and emoticons were also taken into consideration. In addition to manual inspection of these labeled tweets, the tweets were investigated through Bag of Words, top words and their corresponding frequencies for each class, and conjugated words.

**Algorithm 1: polarity assignment to the topic found from the latent Dirichlet allocation Model.**

**Algorithm 1**

- Input: topic list
- Output: aspect for each topic
- /* from nltk.corpus import opinionLexicon
- topicList = Topic List
- sentimentList =
- Topic wise sentiment List */
  - positiveWordFromLexicon = set(opinionLexicon.positive()) /* Positive words from opinionLexicon */
  - negtiveWordFromLexicon = set(opinionLexicon.negative()) /* Negative words from opinionLexico */
  - positiveWords = getTopWords(labeledPositiveTweets) /* Positive words from Positive Labeled tweets */
  - negativeWords = getTopWords(labeledNegativeTweets) /* Negative words from Positive Labeled tweets */
  - ForEach *topic* in *topicList* do
    - positiveCount = 0
    - negativeCount = 0
    - neutralCount = 0
  - ForEach *word* in *topic.WordList* do
    - if (*word* in positiveWordFromLexicon) OR (word in positiveWords) then:
      - positiveCount = 0
    - if (*word* in negtiveWordFromLexicon) OR (word in negativeWords) then:
      - negativeCount += 1
    - if *word* not in (negtiveWordFromLexicon AND negativeWords AND positiveWordFromLexicon AND positiveWords) then:
      - neutralCount += 1
    - if (positiveCount > negativeCount) AND (positiveCount > neutralCount) then:
      - sentimentList[topic:Id] = Positive
    - if (negativeCount > positiveCount) AND (negativeCount > neutralCount) then:
      - sentimentList[topic:Id] = Negative
    - if (neutralCount > positiveCount) AND (neutralCount > negativeCount) then:
      - sentimentList[topic:Id] = Neutral

### Ethics Approval

The research protocol was approved by the School Research Ethics Panel of Allied Health, Faculty of Health, Education, Medicine, and Social Care, Anglia Ruskin University (AH-SREP-19-055).

## Results

The data set was analyzed from the NLP perspective as well as using a qualitative approach.

### Analysis of Unlabeled Data

The analysis of unlabeled data was 2-fold. First, we analyzed the data by converting them into topics using the Gensim LDA Model and exploring the aspects using the NLTK opinion dictionary. Second, we used the VADER model to analyze the data set and define the polarity of each tweet.

From Multimedia Appendix 1, we can see the number of topics and their corresponding coherence values, from which we can observe that the coherence values increase with the number of topics, with the highest value of 0.54 at the index numbers 24, 27, and 29. However, along with the higher coherence values, the lower the number of topics and the lower the overlaps among the topics, the better the topic distribution. The topic distribution visualized in Figure 2 shows that, with the number of topics being 28, the LDA Model overlaps lower than the others. Therefore, we selected the optimal number of the topic index to be 27, which implies that the optimal number of topics in this model index is 28 (the highest coherence is 0.54 with fewer overlaps among the topics) [69]. Moreover, in Figure 2, we show the top 30 salient terms of the data set (on the right) along with the intertopic distance map (on the left).

**Figure 3:**
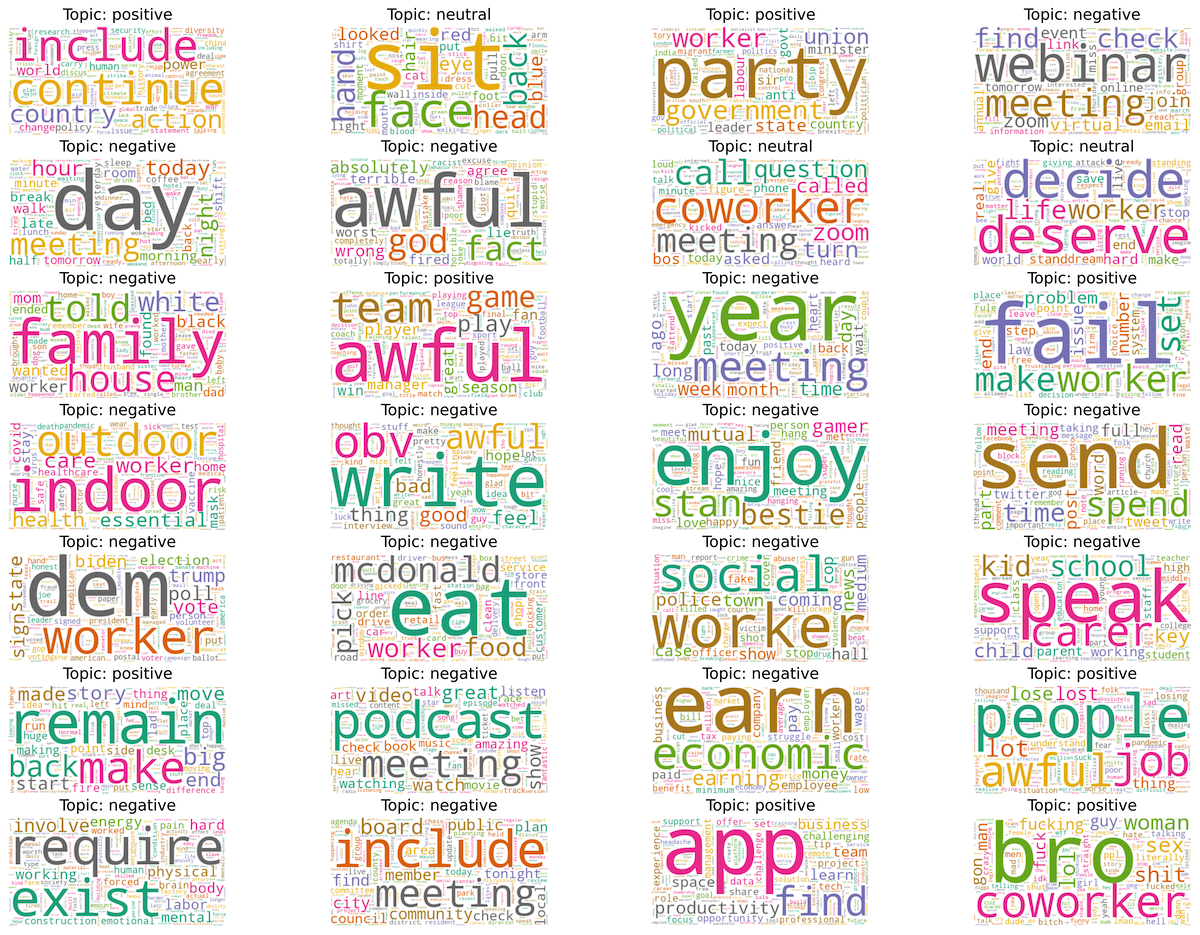
Top words in each topic within the unlabeled data set using latent Dirichlet allocation Mallet.

Figure 3 shows the word cloud of the top words for each topic, along with their broader polarity, where the proportion of positive, negative, and neutral polarity are 28% (8/28), 61% (17/28), and 11% (3/28), respectively. From our analysis, some of the top words from the negative polarity were *awful, bad, lose, lost, tax, pay, money, worker, life, family, social, police, time, meeting, call, care, covid, health, labor, year, week,* and *time*. Many informal (slang) words were also found in the negative topics. The top words from positive polarity were *business, challenge, productivity, zoom, meeting, find, group, app, check, service, food, worker, love, great, amazing, school, child, day,* and *story* and, from neutral polarity, the top words were *vote, poll, coming, town, tonight, game, team, news,* and *today*. Some of the other words from the neutral topics in Figure 3 were *awful, meeting, time,* and *day*. However, similar terms can be used in different contexts, and the topic distribution provides some idea about the contexts of the topics; for example, topic 5 indicates financial aspects, topic 0 indicates web-based meetings and conferences, and topic 7 is about sports.

**Figure 4:**
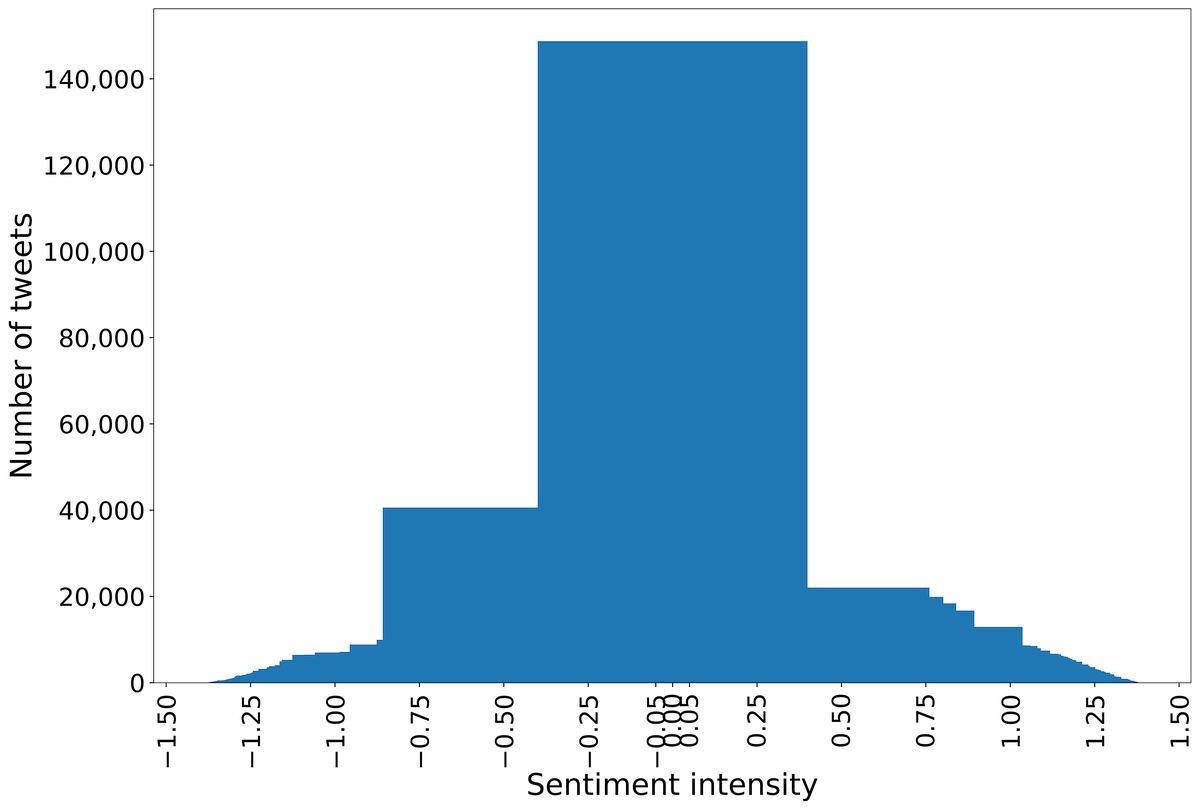
Sentiment intensity of the unlabeled tweets using Valence Aware Dictionary for Sentiment Reasoner.

In the next step, each tweet was analyzed using VADER. It considered the semantic and contextual meanings of a tweet and calculated a value that showed the intensity of the tweet’s sentiment. On the basis of this value, we tagged each tweet as positive, negative, or neutral. In Figure 4, we visualize the number of tweets according to sentiment intensity. Most tweets were within −0.30 to +0.30, which shows the low intensity of negative or positive sentiments. It also shows that a considerable number of tweets expressed a neutral sentiment (intensity value from −0.05 to +0.05).

**Figure 5:**
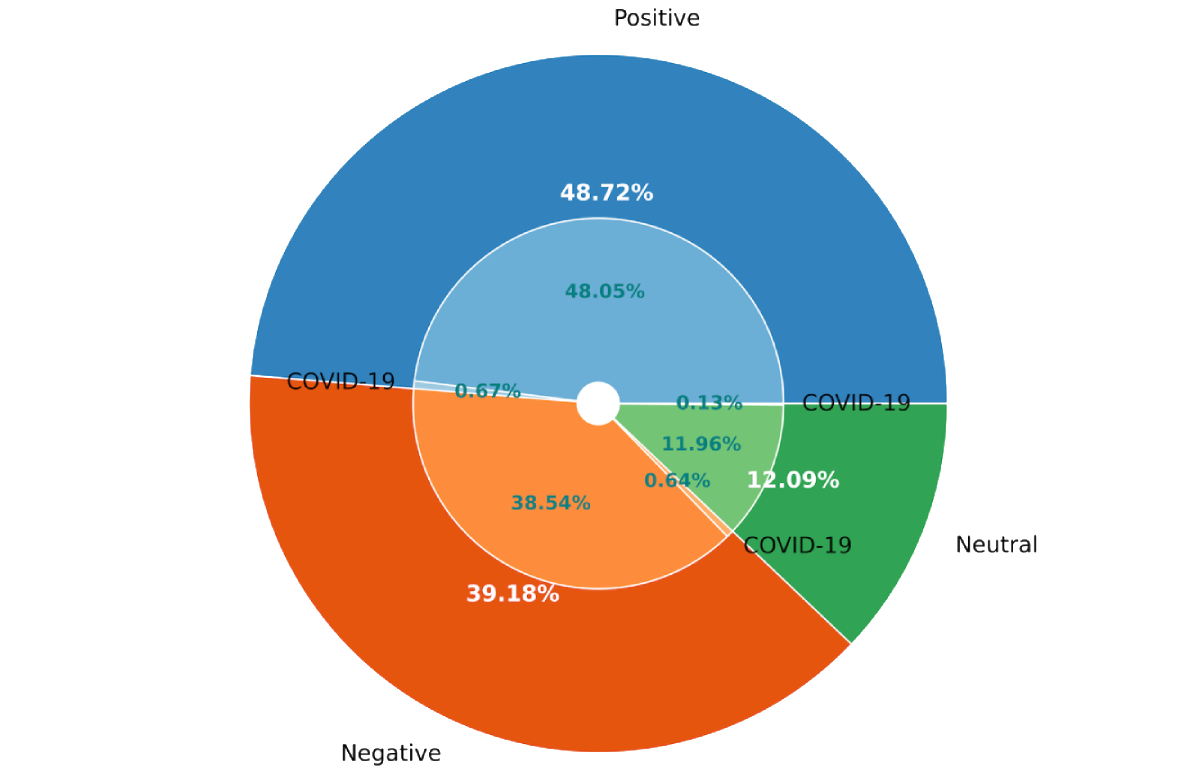
The proportion of positive, negative, and neutral unlabeled tweets using Valence Aware Dictionary for Sentiment Reasoner.

Table 1 shows the intensity level of the tweets, whereas, in Figure 5, we can observe that 48.72% (716,843/1,471,209) of the tweets had positive sentiment, 39.18% (576,462/1,471,209) had negative sentiment, and 12.09% (177,904/1,471,209) indicated neutral sentiments. From Table 1, we observe that most positive tweets have an intensity value between 0.50 and 0.75. The next maximum number of tweets had an intensity between 0.75 and 1.0, which signifies the higher intensity of positive sentiment. By contrast, the maximum number of negative sentiment–showing tweets was within −0.50 to −0.25, which shows the medium level of intensity of negative emotion.

In Figure 5, we also show that 1.44% (21,171/1,471,209) of the tweets mentioned the term “Covid,” among which 0.67% (9854/1,471,209) were positive tweets, 0.64% (9426/1,471,209) were negative tweets, and 0.13% (1891/1,471,209) were neutral tweets.

The negative topics found in the LDA Model were 61% (17/28), and Figure 5 shows that approximately half of the tweets (716,843/1,471,209, 48.72%) expressed positive sentiment. This indicates that, although the negative tweets might be comparatively smaller in number, they imply more diverse subjects than positive tweets relating to work-related mental health based on the analysis conducted using VADER.

**Table 1:** Number of tweets in each quadrant of sentiment intensity.

Group of sentiment intensity	Tweets, n (%)
–1.0 to –0.75	138,659 (9.42)
–0.75 to –0.50	158,974 (10.8)
–0.50 to –0.25	180,014 (12.23)
–0.25 to –0.05	99,028 (6.73)
–0.05 to 0.05	177,691 (12.07)
0.05 to 0.25	105,703 (7.18)
0.25 to 0.50	199,827 (13.58)
0.50 to 0.75	207,439 (14.09)
0.75 to 1.0	203,874 (13.85)

### Analysis of Labeled Data

On the basis of the manually labeled data set, more than half of the tweets (1952/3200, 61%) were categorized or found to be negative; only one-third of the tweets (1088/3200, 34%) expressed the sentiment regarding work-related mental health positively. An exceedingly small portion of the tweets (160/3200, 5%) had an overall neutral sentiment associating work and mental health.

Subsequent to preprocessing and the elimination of the keywords, a digital inspection of this manually labeled data set revealed the 10 most frequent single words and their frequencies for each polarity, as shown in Tables 2, 3, and 4. These figures also show the words that are strongly connected with work or mental health at work either explicitly or implicitly. Certain words, such as *time, like, life, day, get, need,* and *much*, appeared with high frequencies in both positive and negative tweets, which is not surprising linguistically. Therefore, it is important to put these words in context, for which these words (Tables 2, 3, and 4) were put together with their previous or next words in Multimedia Appendices 2, 3, and 4.

**Table 2:** Top 10 frequent words from positive tweets with their corresponding frequencies.

Words	Frequency, n (%)
Time	93 (13.1)
Day	88 (12.5)
Get	81 (11.5)
Life	74 (10.5)
Good	68 (9.6)
Much	64 (9)
Take	63 (9)
Like	60 (8.5)
Year	58 (8.2)
Need	57 (5)

**Table 3:** Top 10 frequent words from negative tweets with their corresponding frequencies.

Words	Frequency, n (%)
Time	153 (13.6)
Like	139 (12.3)
Get	138 (12.1)
Life	136 (12)
Day	117 (10.3)
Much	105 (9.3)
People	88 (7.8)
Need	88 (7.8)
Week	85 (7.5)
One	83 (7.3)

**Table 4:** Top 10 frequent words from neutral tweets with their corresponding frequencies.

Words	Frequency, n (%)
Health	10 (13)
People	9 (11.7)
Get	9 (11.7)
Employee	8 (10.4)
Good	8 (10.4)
Make	7 (9.1)
New	7 (9.1)
Life	7 (9.1)
Year	6 (7.8)
Need	6 (7.8)

For example, time was associated with work and mental health, appearing in both positive and negative tweets. Some of the other words conjugated with time in positive tweets were *vacation, season, lucrative, family, financial, burning, spending, kid, life, care,* and *tough,* whereas, in negative tweets, time was conjugated with words such as *lost, missed, saynotostress (say no to stress), fun, bribed, part time, extra time, hell,* and *full time*. From this digital inspection, it seems that people talk about the time that is related to their *family, vacation, life, financial situation,* or *kid* while positively associating work or mental health, and negative emotions were mostly linked to *part time*, *full time*, or *extra time*. Low income and insecurity about the source of income over time could be some of the underlying reasons for such negative sentiments.

Some other frequent words such as *year* in positive tweets and *week* and *day* in negative tweets also imply time. Some of the words conjugated with year were *parent, people, two years hard, half, best,* and *passed*. In negative tweets, some of the conjugated words were *day long, long traffic, Friday, emotional, delay, project, migraine, packed, insane, blood, exhausted, working week, funeral,* and *unreasonable*. Some slang words were also conjugated in the negative tweets. Analyzing the top words, their adjacent words in terms of meaning, and top words with the conjugated words, time was the most talked about topic concerning work and mental health in the labeled data.

The second most frequent word was *like*. Although it was comparatively less frequent in positive tweets, it was frequent in negative tweets. In positive tweets, some of the conjugated words were *kindness, laughed like never, issue, loss, feeling,* and *death*. In contrast, some of the conjugated words in negative tweets were *feel, diagnosis, opportunity, nothing, terrible, isolated,* and *quite*. The word *feel* was also prevalent in both categories; negative tweets linked feeling with words such as *need, never, fantasy,* and *fresh*. In positive tweets, it was related to words such as *saw, anything, fit,* and *floating*.

*Get* was the third most frequent word in both the positive and negative categories. However, in positive tweets, it was related to words such as *relieved*, *deported*, *break paralegal*, *need mode*, *get done*, *blessed*, *get loved*, and *comfort touch*, whereas, in negative tweets, it was related to words such as *allowing share*, *get rid*, *tonight*, *bad*, *mean*, *grade bad*, *trying sleep*, *enjoy*, *money sad*, and *tension angry*.

*Life* was another frequent word. In positive tweets, it was related to words such as *balance*, *touch*, *goal*, *home*, *personal*, *music*, and *rest*. These imply the issue of work-life balance. Some of the conjugated words in the negative tweets were *personal*, *private*, *entire*, *loss*, *stressful*, *release*, *professionale*, *tough*, *know life lately*, and *normal life bam*.

Positive tweets also contained words such as *good, help,* and *love* with high frequency, whereas negative tweets contained words such as *feel, thing, make,* and *people*.

Neutral tweets mostly contained words such as *healthy, employee, good, make, need, sleep,* and *workplace*, and the most common n-grams of them were *belief healthy behaviour, walkout, difficult people, black people drug, red people, attraction get ticket, break paralegal, employee appreciate, minority, impacted, good, idea make workplace, binge, labour life, intelligence life balance, marriage, weekend, enforcement,* and *break*.

The most frequent words and their n-grams were used to express opposite opinions; this study performed manual qualitative assessments of the labeled tweets. Among the labeled tweets, many claimed that their lifestyle was full of stress—*from dawn to dusk*, *at home*, and *at work*. This observation was in agreement with the n-gram analysis. Similar to the top word analysis, issues of burnout were noticeable during the manual assessment.

For some people, the holidays increase their stress because of the pressure of workload after the holidays—such rationale for this sentiment could not be perceived through the analytical tools.

Commuting from home to the office was found to be stressful for many tweeters, which is also in agreement with the n-gram analysis.

Tweeters often directly communicate their physiological and mental symptoms linked to work, such as *back pain, headache, nervousness, anxiety, sleep deprivation,* and *loss of appetite,* in words. Tweeters attempt to reduce work stress through smoking and substance use, which was evident in the n-gram–based analysis as well.

There were also many tweets only stating work stress using upper case, emphasizing intense stress. The qualitative inspection of this data set revealed work stress in the sporting world, education, and health professions as well as concerning elections, none of which was highlighted through n-gram–based analysis.

Work-home balance and marital and relationship issues were spotted in the labeled tweets, as highlighted by the n-gram analysis as well. High-risk jobs such as firefighting were linked to work stress and marital life. Remarks on work stress and less time spent with spouses, leading to an irritable state of mind, were also noticeable. Tweeters (ie, users of Twitter) also spoke about bad moods as a result of work stress in general. Tweeters expressed confusion regarding the cause and effect of work stress and exhaustion.

Tweeters spoke of suicide, not as direct thoughts or passive suicidal ideation but rather as suicidal incidents associated with stress within their social group. It was not clear whether the person in question belonged to their own work group or professional fraternity or was someone they knew whose suicide was linked to work.

The manual qualitative analysis also revealed some interesting coping mechanisms; for example, being inappropriate in tweet comments was mentioned as one of the coping strategies for work stress. Positive tweets on work-related mental health also mostly concentrated on coping strategies such as listening to music by certain trending artists, gaming, watching a situation comedy on television, traveling, spending time with friends, and social drinking. Tweeters also shared auto-suggestions such as letting go of work stress to prioritize life and a philosophy on impermanent life. The practice of mindfulness and relaxation was encouraged by the positive tweeters to relieve work stress.

Different studies and research findings contributed to most of the neutral tweets in the labeled data set. Owing to the careful selection of the keywords, ambiguity regarding the broader polarity of sentiments was restricted. However, some of the neutral-labeled tweets contained recommendations on how to deal with work stress as they could not be clearly categorized as positive.

Analyzing mood- and mental health–related issues is not a simple task as emotions are often mixed or ambivalent. It is possible to describe mixed emotions as the simultaneous experience of different combinations of opposing emotions, and positive and negative emotions can occur simultaneously, as is evident in this section as well.

## Discussion

### Challenges

In 2019 to 2020, the economic expense of lost working days was estimated to be >GPB 16 billion (US $19.3 billion) [1]. The official numbers suggest an expenditure of GBP 3.5 billion (US $4.2 billion) borne by the UK government (at the end of the day, the UK population) because of work-related injuries and ill health [1]. Ignoring such prevalence of work-related poor health adds more to the national disease burden, which may not be reflected as a direct cost occurring from the unhealthy and toxic working conditions. In 2018 to 2019, employers faced a loss of GBP 3.2 billion (US $3.9 billion) as workers were off sick for reasons attributable to work itself [1]. The pandemic and the socioeconomic consequences in the postpandemic period have created a more unsettling environment. An unhappy workforce hinders the overall growth and productivity of any organization [70,71]. However, most of the economic burden of work-related mental and physical ill health is borne by the worker and their friends and family, who also bear the emotional hardship of such adversity. Therefore, breaking the cycle of poor health outcomes is a point of interest at a national, organizational, and personal level.

Nowadays, mental health and well-being have become new buzzwords, which can be thought of as both positive and negative signs. Recognizing the problem is always the first step. More visibility and open discussions around mental health issues may open the door to reducing stigma and breaking the taboo on this subject. Self-awareness and strategic approaches from the employer may significantly reduce the mental health effects arising because of the workplace. Many commercial and nonprofit organizations have been trying to pave their way into this market by delivering business-to-business support, such as Minddistrict [72], the Health Assured employee assistance program [73], and Nuffield Health [74].

AI-enabled, data-driven approaches have already shown promising results in predicting quitters in the workforce [75]. Zegami [76], launched at the University of Oxford, has been working on a tool to identify unhappy employees using a wide range of data such as age, salary, benefits, and work location [77].

The sentiment around the workplace may be expressed in words, body language, physiological parameters, acts and actions, or responses to others. However, it can be challenging to prove the root cause of the issue when the background narrative is also part of the problem—psychological, socioeconomic, cultural, and person-specific profiles may define how such sentiment is perceived and expressed. Moreover, one-third of work-related ill health is due to musculoskeletal disorders [1]. There is a consensus among clinicians and occupational health experts that musculoskeletal disorders are linked to mental health issues, the mechanism of which is yet to be unraveled [78,79]. Work-related ill health, linked to psychosomatic effects, cultivates a vicious cycle of habits, such as work-related anxiety and stress causing insomnia, which causes headache and increased intake of stimulants, which causes more sleepless nights, which causes stress, and the cycle continues.

### Framework for Combining Existing Technologies

**Figure 6:**
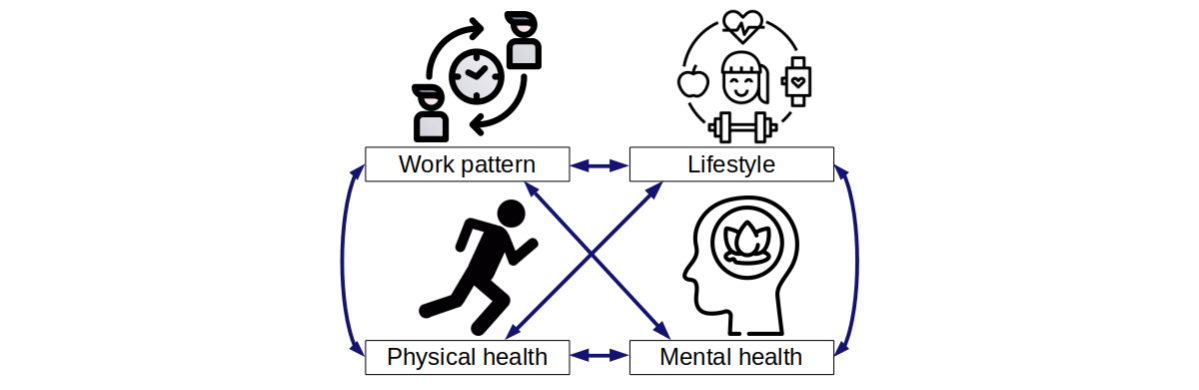
Dynamic relationship between work life and health.

The complex and dynamic relationships (Figure 6) among work patterns, lifestyle, individualized work patterns, occupational hazards, health, and well-being are all intertwined with the workplace sentiments. Harnessing the power of AI and big data analytics, a descriptive model can be constructed to unravel the shared responsibility among stakeholders (eg, the individual, employer, and industry).

**Figure 7:**
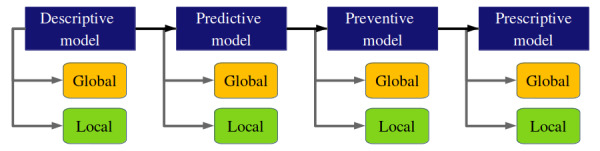
High-level architecture of artificial intelligence–enabled mental health support for the workforce.

This study sketches a framework to understand the vulnerable zone of work-related sentiments to facilitate better work practice and health outcomes (Figure 7). In Figure 7, the global descriptors within the problem space can be broadly categorized into three domains as follows: (1) industry-specific elements arising from the nature of the occupation (eg, clinicians at night shifts); (2) organization-specific workplace culture (eg, social drinking culture); and (3) societal descriptors such as the Gross Happiness Index, political stability, crime rate, employment rate, living expenses, economy, social inclusion, diversity, and tolerance, the data for which can be aggregated from national and global databases. These global descriptors directly influence the local descriptors forming an unbreakable pattern; however, the impact can vary widely from person to person.

Developing an understanding of how individuals feel about such external variables may not be reflected in numbers (see the Introduction section) or explicitly expressed in words (see this section). Deeper insights into the factors responsible for work-related sentiments through the local descriptor need to be coproduced by engaging the workforce in the process. Cross-referencing the local descriptors of the descriptive model with real-time lifestyle tracking along with global descriptors of mental health disorders (data to be collected through electronic health records) can inform AI-enabled predictive models. Evidence-based prediction will empower stakeholders to make informed decisions, design appropriate preventive measures to reduce the prevalence of work-related ill health, and introduce effective interventions to keep the workforce healthy and happy.

### The Discourse Regarding Ultrasmart Sentiment Detection

The habit and necessity to share personal information on public platforms and the control over the outcome of sharing such information are 2 sides of the same coin in the era of datafication. The consequence of carefree users relinquishing rights to privacy increases the risk of invasion of privacy of users who are not even engaged with the process. The end users are often unaware of digital traps such as “Cookies” and “personalization” and the emerging and inconceivable powers of AI with invisible data links, resulting in an accidental discovery of an untold (fabricated) story and data breaches. Such an outcome often results from the negligence of the data controller and data processor. Examples are evident in the literature [80-82].

**Figure 8:**
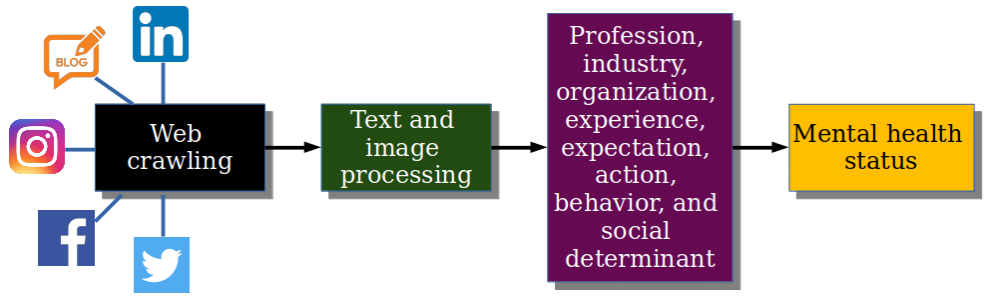
Self-imposed digital surveillance without a security breach.

This study presents a hypothetical data map of how publicly available disintegrated data can reveal the “digital” mental health of an employee (Figure 8). An employer can analyze the time-stamped digital footprint to evaluate and predict the performance of employees; however, it is questionable without full consent on data collection and processing and without strong data governance and data ethics in place. Such profiling, as shown in Figure 8, can be constructed in a third-party organization or even via bots. Although the General Data Protection Regulation protects such personal data mining and processing up to a certain degree within the United Kingdom and European Union region, care should be taken to safeguard the vulnerable workforce, which is already experiencing poor mental health conditions. Although this study advocates for a more comprehensive approach using a wide range of technological tools to better perceive the occupational health concerns and improve the health and well-being of the workforce, care should be taken while using these emerging techniques to avoid the creation of a Pandora’s box of digital surveillance.

### Research Limitations and Future Work

Twitter is one of the most popular microblogs for researchers, where millions of people express their feelings. Owing to its character limit, users are bound to write specific data, which helps in analyzing them. Moreover, Twitter provides application programming interfaces for researchers to fetch and analyze archived tweets in an enormous number [83]. However, not all working adults are on social media, not all tweeters are verbally expressive, and a single event of microblogging can be insufficient and inconclusive to perceive deeper emotions. Moreover, millions of users prefer other microblogging sites to express their emotions. Therefore, despite having many advantages for researchers, using Twitter alone as a data source is a limitation. Although this study considered public tweets, the research was subject to approval from one of the internal ethics committees. They provided approval on the grounds that tweets could not be directly quoted on the paper, and no tweeter would be tracked. Therefore, we could not include and analyze any personal data such as usernames and locations. A more detailed context for the inclusion of such data can be found elsewhere [84-86]. However, this study used an extensive data fetching process to avoid such limitations. Furthermore, considering the ethical limitations, a case study will require us to recruit participants, which needs to be carefully done as a constant awareness of being analyzed may hamper the spontaneity of the tweets. The inclusion of geographical analysis and longitudinal observation of the same tweeter in the future can provide richer insights and a better context of work-related sentiments to unveil a pattern, if it exists. However, data governance in data processing should be prioritized, and constant caution should be exercised so that users’ privacy is not hampered. Although including a case study and geographical analysis is beyond the scope of this study, we are encouraged to do so in the future.

### Conclusions

This paper presented a synopsis of the current state of work-related mental health, its ripple effect beyond working hours, and how it is being dealt with. The examination of >1 million tweets in the aforementioned sections shows distinct aspects of work-related sentiments that people are willing to share on social media. The key findings of this study are outlined in Textbox 6.

Analyzing thousands of tweets, this study exposed the aspects that the workforce is concerned about, which are in agreement with the LFS survey to some extent. However, the subject matter requires a more integrated approach. Moreover, irony, sarcasm, emojis, multipolarity, and word ambiguity are some of the challenging aspects of the NLP domain that require further enhancement. Therefore, we emphasize exploring how technology-driven solutions can support the unraveling of the dynamically intertwined relationship among work, mental and physical health, and lifestyle to protect our workforce and transform the workplace.

**Key findings of this study.**

**Key findings**

- The type of words people use to express work-related sentiments were identified.
- Colloquialism and eloquence were both observed in Twitter expressions regarding work, whether expressing grief or happiness.
- An overlapping set of words in both positive and negative tweets signifies upbeat and, by contrast, undesirable feelings affecting the same entities (day, life, and time) in their lives.
- Time variables (*day*, *time*, and *week*), *life*, and *need* were the predominant words in both positive and negative tweets—as is evident from the trigrams. The topic analysis provided a sense of contextual narrative across sentiments. For example, topic 5 indicates financial aspects, topic 0 indicates internet-based meetings and conferences, and topic 7 is about sports.

## Appendix

Multimedia Appendix 1 Coherence values of the latent Dirichlet allocation Model for a number of topics from 2 to 31.

Multimedia Appendix 2 Top words from positive tweets with previous and next words (trigrams).

Multimedia Appendix 3 Top words from negative tweets with previous and next words (trigrams).

Multimedia Appendix 4 Top words from neutral tweets with previous and next words (trigrams).

## Abbreviations

AI: artificial intelligence
GloVe: Global Vectors for Word Representation
HSE: Health and Safety Executive
LDA: latent Dirichlet allocation
LFS: Labour Force Survey
NLP: natural language processing
NLTK: Natural Language Toolkit
VADER: Valence Aware Dictionary for Sentiment Reasoner
