# Supplementary material for: Thinking Aloud or Screaming Inside: Exploratory Study of Sentiment Around Work
Source: JMIR Form Res. 2022 Sep 30;6(9):e30113. doi: 10.2196/30113 (PMC9568814; doi:10.2196/30113)
Supplement: Multimedia Appendix 1 [file formative_v6i9e30113_app1.pdf]

Multimedia Appendix 1. Coherence values of LDAModel for a number of Topics from 2 to 31.

Topic Number	Coherenc e
2	0.32
3	0.4
4	0.42
5	0.45
6	0.47
7	0.48
8	0.49
9	0.49
10	0.5
11	0.5
12	0.51
13	0.52
14	0.52
15	0.53
16	0.52

Topic Number	Coherenc e
17	0.52
18	0.52
19	0.53
20	0.53
21	0.53
22	0.53
23	0.53
24	0.54
25	0.53
26	0.53
27	0.54
28	0.52
29	0.54
30	0.53
31	0.53
