# Supplementary material for: Thinking Aloud or Screaming Inside: Exploratory Study of Sentiment Around Work
Source: JMIR Form Res. 2022 Sep 30;6(9):e30113. doi: 10.2196/30113 (PMC9568814; doi:10.2196/30113)
Supplement: Multimedia Appendix 2 [file formative_v6i9e30113_app2.pdf]

Multimedia Appendix 2. Top words from Positive tweets with previous and next word (trigrams)

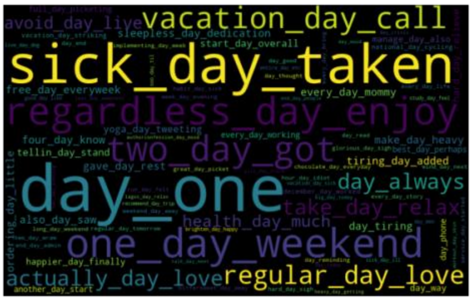

S1: day

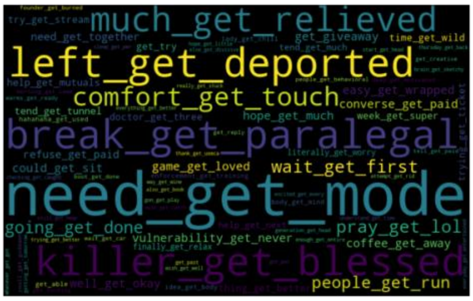

S2: get

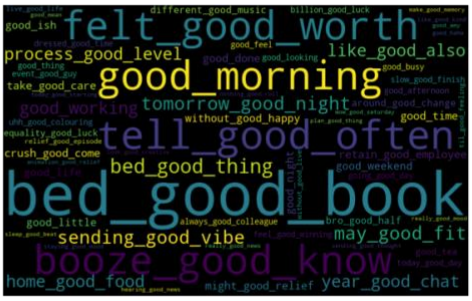

S3: good

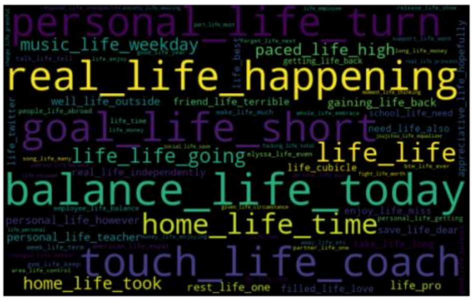

S4: life

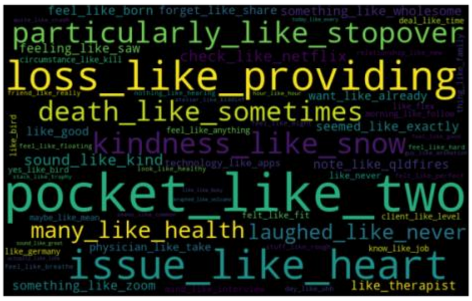

S5: like

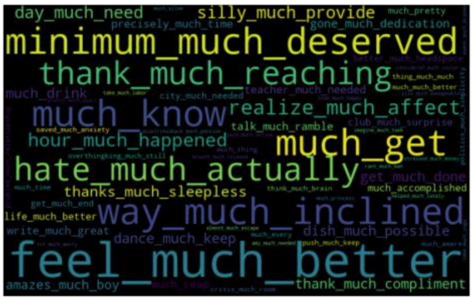

S6: much

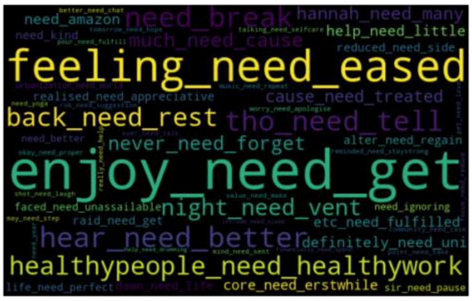

S7: need

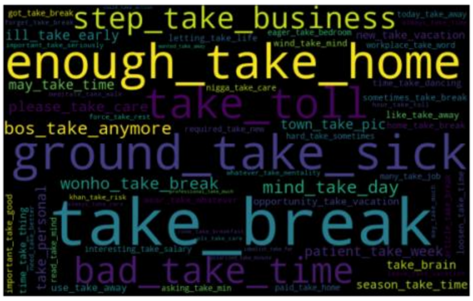

S8: take

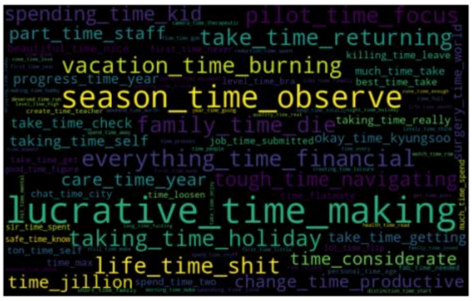

S9: time

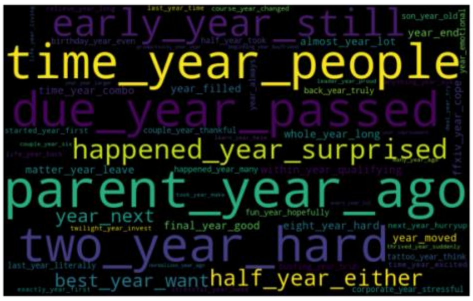

S10: year
