# Supplementary material for: Thinking Aloud or Screaming Inside: Exploratory Study of Sentiment Around Work
Source: JMIR Form Res. 2022 Sep 30;6(9):e30113. doi: 10.2196/30113 (PMC9568814; doi:10.2196/30113)
Supplement: Multimedia Appendix 3 [file formative_v6i9e30113_app3.pdf]

Multimedia Appendix 3. Top words from Negative tweets with previous and next word (trigrams)

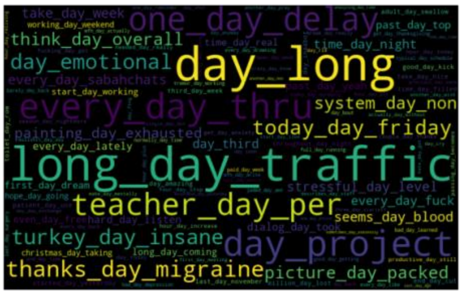

S11: day

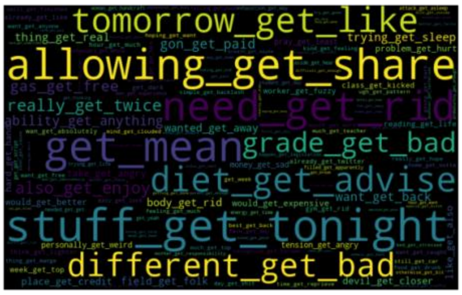

S12: get

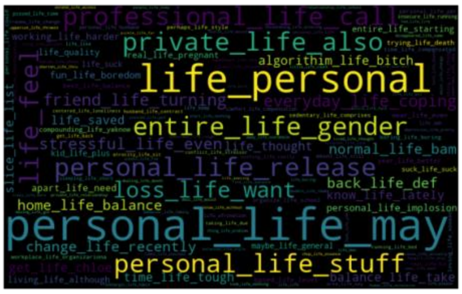

S13: life

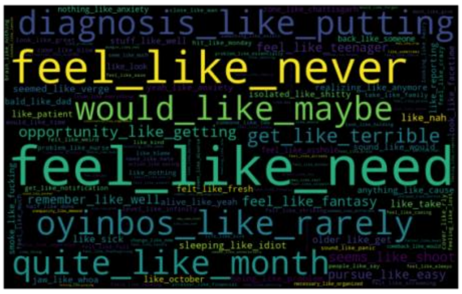

S14: like

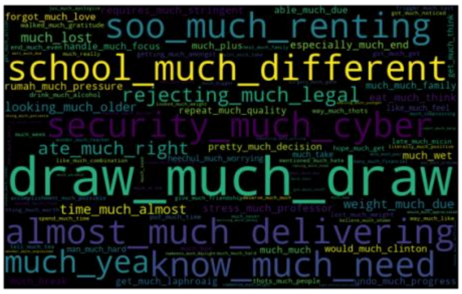

S15: much

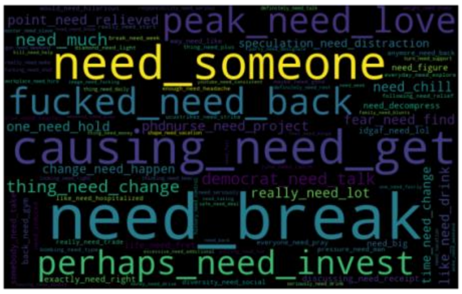

S16: need

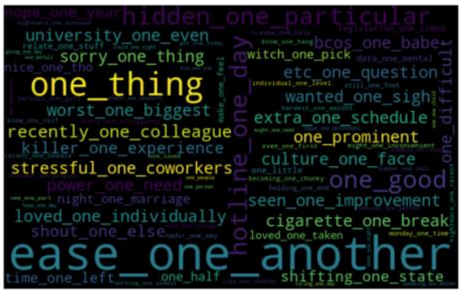

S17: one

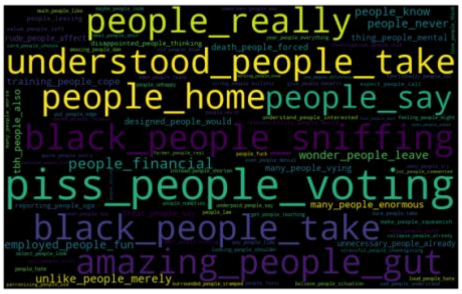

S18: people

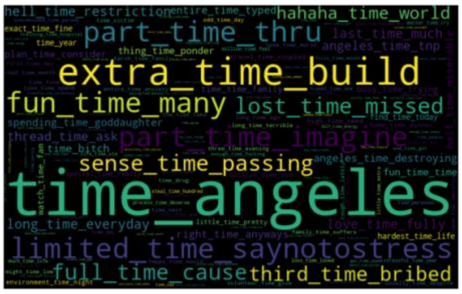

S19: time

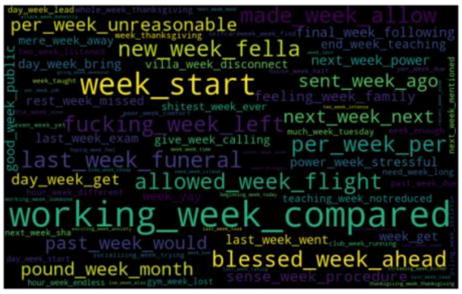

S20: week
