# Supplementary material for: Thinking Aloud or Screaming Inside: Exploratory Study of Sentiment Around Work
Source: JMIR Form Res. 2022 Sep 30;6(9):e30113. doi: 10.2196/30113 (PMC9568814; doi:10.2196/30113)
Supplement: Multimedia Appendix 4 [file formative_v6i9e30113_app4.pdf]

Multimedia Appendix 4. Top words from Neutral tweets with previous and next word (trigrams)

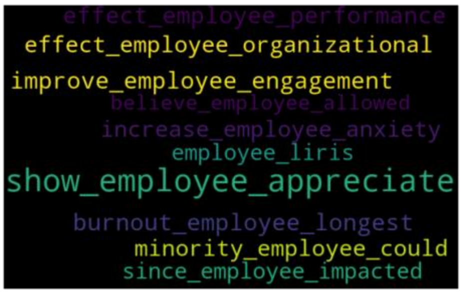

S21: employee

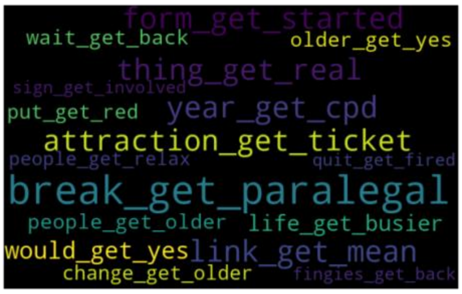

S22: get

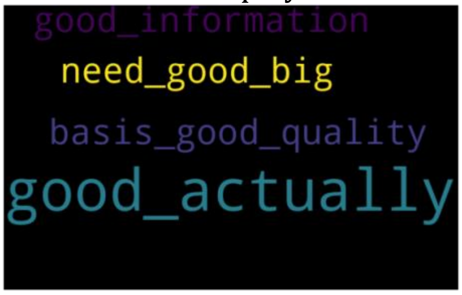

S23: good

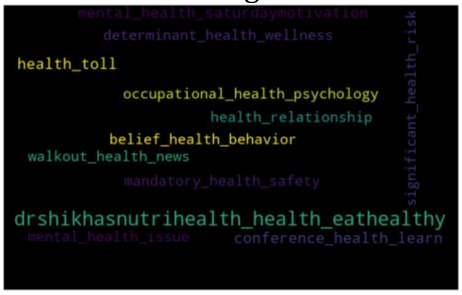

S24: health

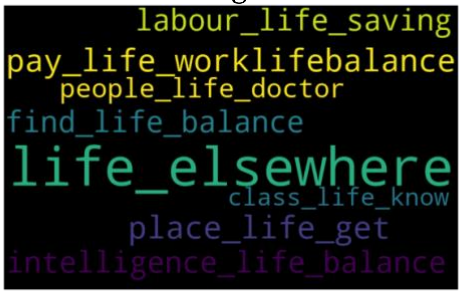

S25: life

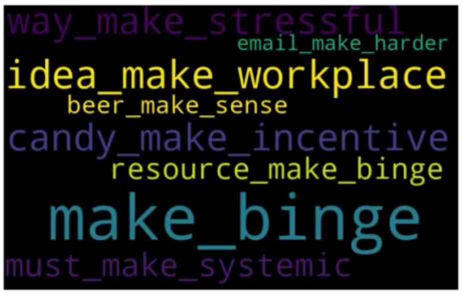

S26: make

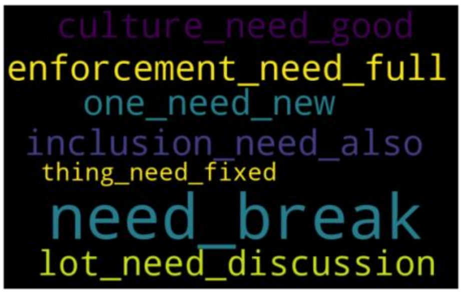

S27: need

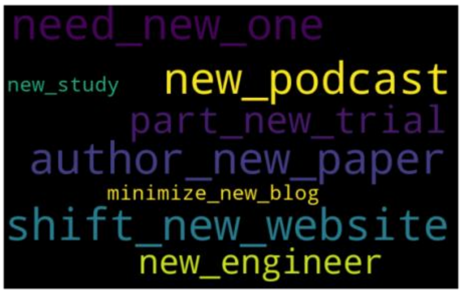

S28: new

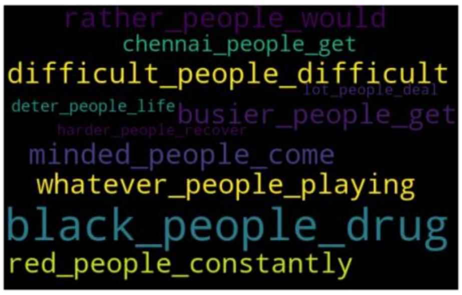

S29: people

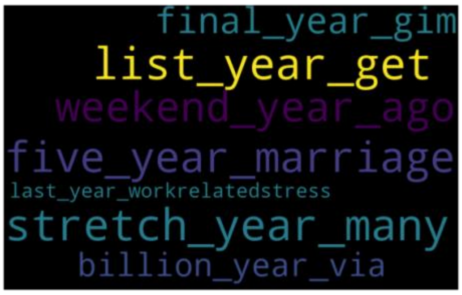

S30: year
